# Fungicidal Activity in the Presence of Keratin as an Important Factor Contributing to In Vivo Efficacy: A Comparison of Efinaconazole, Tavaborole, and Ciclopirox

**DOI:** 10.3390/jof3040058

**Published:** 2017-10-19

**Authors:** Haruki Tachibana, Naomichi Kumagai, Yoshiyuki Tatsumi

**Affiliations:** Pharmacology Department, Drug Research Center, Kaken Pharmaceutical Co., Ltd. 14, Shinomiya, Minamigawara-cho, Yamashina-ku, Kyoto 607-8042, Japan; kumagai_naomichi@kaken.co.jp (N.K.); tatsumi_yoshiyuki@kaken.co.jp (Y.T.)

**Keywords:** onychomycosis, topical antifungal, drug effect, keratin, fungicidal activity

## Abstract

Use of oral antifungals in the treatment of onychomycosis is commonplace; but their use can be limited by safety and patient concerns. Due to their broader safety margins, topical antifungals (efinaconazole, tavaborole, and ciclopirox) are a useful option in the treatment of mild-to-moderate onychomycosis in the USA, but their antifungal activity has yet to be directly compared. This study aims to identify important factors contributing to in vivo efficacies of the three topical antifungals. Minimum inhibitory concentrations (MICs) were determined by Clinical and Laboratory Standards Institute (CLSI) M38-A2 broth microdilution. The MIC_90_ values of efinaconazole, tavaborole, and ciclopirox for *T. rubrum* were 0.0078, 8.0, and 0.50 μg/mL, respectively. The MIC_90_ values for *T. mentagrophytes* were 0.016, 8.0, and 0.50 μg/mL, respectively. Efinaconazole showed potent fungicidal activity in keratin-containing medium, whereas tavaborole was fungistatic, and ciclopirox not active. In the guinea pig model of onychomycosis, the therapeutic efficacy of efinaconazole was superior to those of tavaborole and ciclopirox. This study suggests that not only fungistatic activity (MIC), but also fungicidal activity in the presence of keratin, is an important factor contributing to the in vivo efficacy of topical antifungal drugs against onychomycosis.

## 1. Introduction

**Figure 1:**
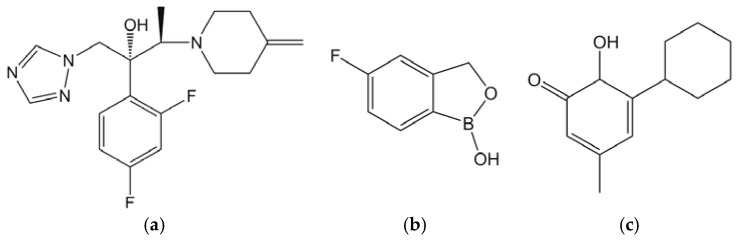
Chemical structures of efinaconazole (**a**), tavaborole (**b**), and ciclopirox (**c**).

Onychomycosis is a common fungal nail infection, mainly caused by *Trichophyton rubrum* (*T. rubrum*) and *Trichophyton mentagrophytes* (*T. mentagrophytes*) in the nail plate and nail bed. Prevalence of onychomycosis has been estimated at between 10% (Japan) and 13.8% (USA) [1,2]. Onychomycosis impacts the patient’s quality of life (QOL) due to walking difficulties and the poor appearance of the nail, and can be a source of secondary infection or spread to other family members. Several antifungals, such as oral itraconazole, oral terbinafine, topical amorolfine nail lacquer, and topical ciclopirox nail lacquer, have been used to treat onychomycosis. In general, oral treatment with itraconazole and terbinafine has been shown to be more effective with high complete cure rates of 26% and 55%, respectively [3], but they have the disadvantage of drug-drug interactions and systemic side effects (e.g., hepatotoxicity) [4]. On the other hand, although topical treatment with amorolfine or ciclopirox nail lacquer is not generally associated with systemic side effects due to their extremely low transition from the nail to the bloodstream, they appear to be less effective, with complete cure rates of 0.96% and 5.5–8.5%, respectively [5,6]. Two topical antifungals, efinaconazole (Figure 1a) and tavaborole (Figure 1b), were recently launched in the USA. Complete cure rates with efinaconazole and tavaborole in two clinical trials were 15.2–17.8% and 6.5–9.1%, respectively [7,8]. The complete cure rates with efinaconazole were higher than those previously reported for ciclopirox (Figure 1c) and tavaborole, and were similar to those reported for oral itraconazole [7,9]. Efinaconazole has demonstrated superior in vivo efficacy to amorolfine and ciclopirox, due to its better nail permeation and lower MIC [10,11]. Tavaborole has also been shown to have lower antifungal activity than ciclopirox, but a higher in vitro antifungal activity against dermatophytes under the nail plate in the TurChub^®^ system, because of its better nail permeation [12]. We are not aware of any comparisons of the in vitro and in vivo antifungal activities of efinaconazole and tavaborole. In the present study, we use three antifungals with different modes of action (efinaconazole, tavaborole, and ciclopirox) [13], and investigate whether fungicidal activity in the presence of keratin or MIC-determined CLSI method influences the efficacy of topical antifungal drugs using a guinea pig onychomycosis model.

## 2. Materials and Methods

### 2.1. Test Substances

Efinaconazole was obtained from Kaken Pharmaceutical Co., Ltd. (Kyoto, Japan). Tavaborole was purchased from Toronto Research Chemicals Inc. (Toronto, ON, Canada). Ciclopirox was purchased from Sigma-Aldrich (St. Louis, MO, USA). Efinaconazole 10% (*w*/*w*) solution was purchased from Valeant Pharmaceuticals International Inc. (Laval, QC, Canada). Tavaborole 5% (*w*/*w*) solution and Ciclopirox 8% (*w*/*w*) nail lacquer were purchased from Pharmaderm (Princeton, NJ, USA) and G&W Laboratories Inc. (South Plainfield, NJ, USA), respectively.

### 2.2. Media and Keratin

Sabouraud dextrose agar (SDA) powder was purchased from Becton, Dickinson and Company (Franklin Lakes, NJ, USA). Potato dextrose agar (PDA) powder, brain heart infusion agar (BHIA) powder, Roswell Park Memorial Institute (RPMI) 1640 medium powder, and lecithin from soy bean were purchased from Nissui Pharmaceutical Co., Ltd. (Tokyo, Japan). Glucose peptone agar with lecithin and polysorbate 80 (GPLP) was purchased from Nihon Pharmaceutical Co., Ltd. (Tokyo, Japan). 3-(*N*-morpholino)propanesulfonic acid (MOPS)-buffered RPMI 1640 medium was prepared according to the guidelines of CLSI M38-A2. Modified GPLP agar plate containing 1% lecithin, 10 μg/mL of chloramphenicol, 500 μg/mL of cycloheximide, 50 μg/mL of 5-fluorocytosine, and 75 μg/mL of gentamycin was used to isolate dermatophytes from the nails of guinea pigs. Cycloheximide and 5-fluorocytosine were used to prevent contamination of fungi, excluding dermatophytes; and chloramphenicol and gentamycin were used to prevent contamination of bacteria.

Porcine hoof was used as a substitute for human nail for preparing keratin powder, because several antifungals have similar affinities for porcine hoof and human nail keratins [10,14]. Hasuko et al. have also suggested the versatility of porcine hoof powder as an alternative to human keratin preparation for non-clinical study [15]. Porcine hooves (OC farm, Hokkaido, Japan) were powdered using a Wonder Blender (Osaka Chemical Co., Ltd., Osaka, Japan) and then defatted by diethyl ether and ethanol mixture (1:1, *v*/*v*). This powder was sterilized by autoclaving at 121 °C for 15 min.

### 2.3. Test Organisms

*T. rubrum*: strains NBRC 5808 and NBRC 6204 were obtained from the National Institute of Technology and Evaluation (NITE, Chiba, Japan); strains IFM 46615, IFM 47615, IFM 47618, IFM 47623, IFM 47625, IFM 47629, and IFM 46157 were obtained from the Medical Mycology Research Center, Chiba University (Chiba, Japan); and strains ATCC MYA-4438 and ATCC 18759 were obtained from the American Type Culture Collection (Manassas, VA, USA). *T. mentagrophytes*: strains IFM 47176, IFM 47179, IFM 48798, IFM 48803, IFM 48805, IFM 52442, and IFM 55366 were obtained from the Medical Mycology Research Center, Chiba University (Chiba, Japan); strain ATCC MYA-4439 was obtained from the American Type Culture Collection (Manassas, VA, USA); and strains SM-110 and KD-04 were gifted from Niigata University School of Medicine (Niigata, Japan) and Teikyo University School of Medicine (Tokyo, Japan), respectively.

### 2.4. MIC Study of Antifungals for T. rubrum and T. mentagrophytes

The MICs of efinaconazole, tavaborole, and ciclopirox for 10 strains of *T. rubrum* and 10 strains of *T. mentagrophytes* were measured according to the CLSI M38-A2 broth microdilution method. Each test substance was dissolved in dimethyl sulfoxide (DMSO) to prepare 6.4 mg/mL solution. Two-fold serial dilutions were prepared, and these were further diluted 50-fold by using MOPS-buffered RPMI 1640 medium. A total of 100 μL of this solution was applied onto 96 well microplate; then, 100 μL of MOPS-buffered RPMI 1640 medium, containing 4 × 10^3^ microconidia/mL of test organisms, was added (final fungal concentration: 2 × 10^3^ microconidia/mL). The microplates were incubated at 35 °C for 4 days. After being incubated, the MIC of each drug was measured as the lowest concentration at which the test substance exerted approximately 80% inhibition against the growth of the test organism from the growth control by visual reading. MIC_50_ and MIC_90_, the lowest test substance concentrations at which 50% and 90% of the test strains were inhibited, were calculated for each drug.

### 2.5. Time-Kill Study of Antifungals against T. mentagrophytes in the Presence of Keratin

Each antifungal was dissolved in DMSO (2 mg/mL). Four-fold serial dilutions were prepared and these further diluted 50-fold using MOPS-buffered RPMI 1640 medium. A total of 250 μL of this solution was added to 100 mg of sterilized keratin powder. Two-hundred and fifty microliters of MOPS-buffered RPMI 1640 medium, containing 2 × 10^4^ microconidia/mL of *T. mentagrophytes* strain SM-110, was added to the drug-keratin mixture (final fungal concentration: 1 × 10^4^ microconidia/mL), and cultured at 35 °C for 3, 7, 10, and 14 days. After being incubated, culture medium was collected and homogenized with a glass homogenizer. One hundred microliters of this homogenate was spread onto a GPLP agar plate, and incubated at 30 °C for 14 days. Colonies that appeared on the plates were counted, and the logarithmic value of colony forming units/mL (Log CFU/mL) and their standard deviations (SD) in the culture medium calculated.

### 2.6. Therapeutic Efficacy of Topically Applied Antifungals in a Guinea Pig Onychomycosis Model

Kaken Pharmaceutical Co., Ltd. follows in-house regulations in complying with “Japan’s Act on Welfare and Management of Animals”, and the related international and domestic guidelines. The Institutional Animal Care and Use Committee of Kaken Pharmaceutical Co., Ltd. reviews whether all animal experimental protocols are prepared based on the “3Rs (Replacement, Reduction and Refinement) principle” in advance, and implements self-inspections and assessments of the animal experiment processes and the facility operations. This study was approved by the committee on 18 August 2015, with the identification code of this study being (K15-165).

This study was performed according to the previously described method [10]. For arthrospore formation, *T. mentagrophytes* strain SM-110 was cultured on BHIA plate at 30 °C for 10 days in 18% CO_2_ containing air. The hind-paw nails of six-week-old male Hartley strain guinea pigs (Japan SLC, Inc., Hamamatsu, Japan) were infected with arthrospores (1 × 10^7^ cells/foot) of *T. mentagrophytes* for four weeks. It is reported that tinea pedis caused by strain SM-110 are histologically similar to human tinea pedis [16], and the strain invades the nail plate [17] just as *T. rubrum* does human nail plate. After the onychomycosis model was produced, animals were assigned to four groups of six animals each. Thirty microliters of efinaconazole 10% solution, tavaborole 5% solution, or ciclopirox 8% nail lacquer was topically applied to the nails once daily for four weeks. In reference to the directions for clinical use, the nail surface treated with ciclopirox 8% nail lacquer was wiped with absorbent cotton containing 70% (*v*/*v*) ethanol once every week before the test substance was applied. There was an untreated infected control group. The day after the final treatment, all animal nails were wiped with absorbent cotton containing 70% ethanol to remove any residual formulation. After one week, animals were sacrificed and nails collected from the feet. Nails were minced thoroughly, and nail powder homogenized in phosphate-buffered saline containing 0.25% (*w*/*v*) trypsin and 10 mmol/L FeCl_2_. The homogenates were incubated at 37 °C for an hour. One hundred microliters of homogenate was spread onto the modified GPLP agar plates containing antibiotics and lecithin. These plates were cultured at 30 °C for 14 days. After incubation, colonies that appeared on the plates were counted, and the mean values of Log CFU/foot and their SDs were calculated. Fungal cell counts in each group were compared using Tukey’s multiple comparison tests in EXSUS (CAC croit corporation, Tokyo, Japan). A *p*-value of less than 0.05 was regarded significant.

## 3. Results

### 3.1. MIC Study of Antifungals for T. rubrum and T. mentagrophytes

**Figure 2:**
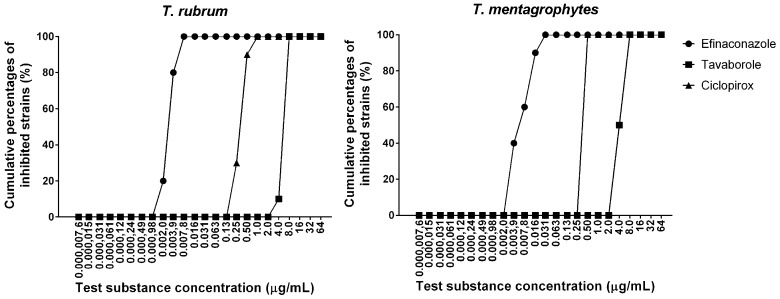
Cumulative MIC frequency distribution of efinaconazole, tavaborole, and ciclopirox for *T. rubrum* and *T. mentagrophytes* (each 10 strain).

The MICs of efinaconazole, tavaborole, and ciclopirox for *T. rubrum* were in the ranges of 0.0020–0.0078 μg/mL, 4.0–8.0 μg/mL, and 0.25–1.0 μg/mL, respectively. The MIC ranges for *T. mentagrophytes* were 0.0039–0.031 μg/mL, 4.0–8.0 μg/mL, and 0.50 μg/mL, respectively. The MIC_50_ (MIC_90_) values of efinaconazole, tavaborole, and ciclopirox for *T. rubrum* were 0.0039 (0.0078), 8.0 (8.0), and 0.50 (0.50) μg/mL, respectively. The MIC_50_ (MIC_90_) values for *T. mentagrophytes* were 0.0078 (0.016), 4.0 (8.0), and 0.50 (0.50) μg/mL, respectively (Table 1). The susceptibilities of the two dermatophytes to each drug were similar (Figure 2).

### 3.2. Time-Kill Study of Antifungals against T. mentagrophytes in the Presence of Keratin

**Figure 3:**
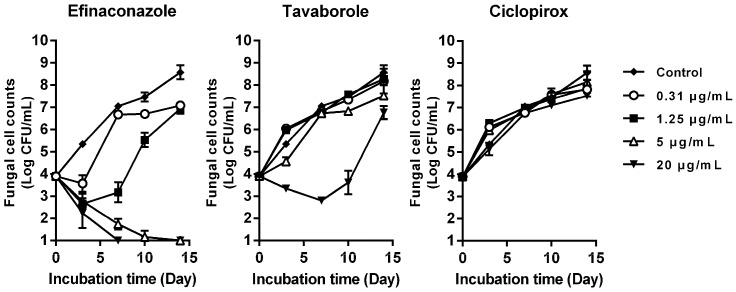
Fungicidal activities of efinaconazole, tavaborole, and ciclopirox in the presence of keratin. *T. mentagrophytes* and drugs were cultured in keratin containing medium at 35 °C for 3, 7, 10, and 14 days, and viable fungal cells were measured by plate count method. Symbols and bars indicate the mean of Log CFU/mL (*n* = 3) and the SD of Log CFU/mL (*n* = 3), respectively. DMSO was used as a growth control.

Time-kill curves of the three antifungals against *T. mentagrophytes* in the presence of keratin are shown in Figure 3. Efinaconazole exhibited potent fungicidal activity against *T. mentagrophytes* at 5 μg/mL and 20 μg/mL in a time-dependent manner, resulting in complete mycological eradication on Day 14 and Day 7, respectively. Tavaborole exhibited only fungistatic activity until Day 10, even at 20 μg/mL, and the fungus then regrew. Ciclopirox was not active at any of the concentrations tested.

### 3.3. Therapeutic Efficacy in a Guinea Pig Onychomycosis Model

**Figure 4:**
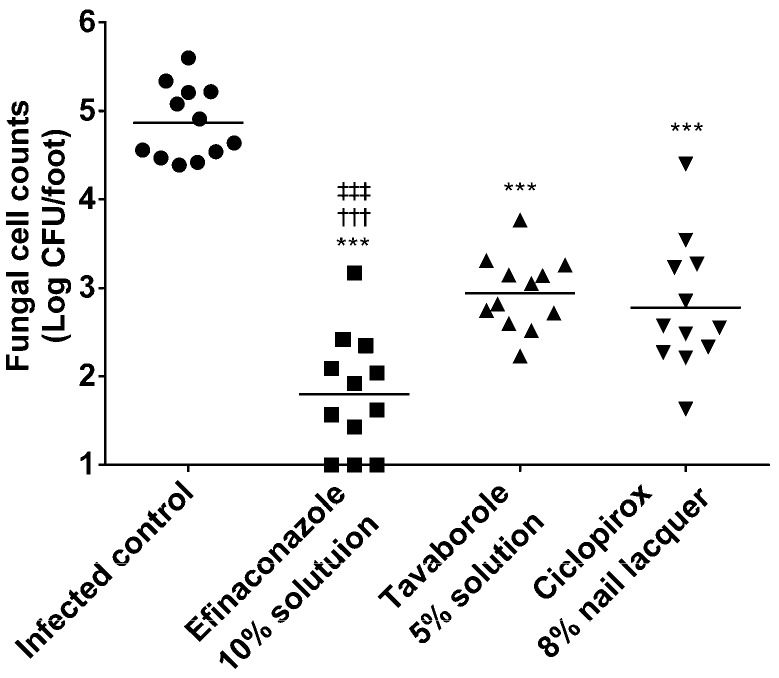
In vivo therapeutic efficacies of efinaconazole 10% solution, tavaborole 5% solution, and ciclopirox 8% nail lacquer in a guinea pig onychomycosis model. Statistical significance was analyzed by Tukey type multiple comparison test. ***: *p* < 0.001 vs. the infected control group, ^†††^: *p* < 0.001 vs. the tavaborole 5% solution group, ^‡‡‡^: *p* < 0.001 vs. the ciclopirox 8% nail lacquer group. Dots: individual data. Bars: the mean of Log CFU/foot (*n* = 12).

Fungal cell counts in the nails of each animal group are shown in Figure 4. Viable cell counts (Log CFU/foot) in the nails of the infected control, and those treated with efinaconazole 10% solution, tavaborole 5% solution, or ciclopirox 8% nail lacquer were 4.87 ± 0.41, 1.80 ± 0.66, 2.94 ± 0.42, and 2.78 ± 0.73 (mean ± SD, *n* = 12), respectively. For all antifungals, viable cell counts were significantly lower than in the infected control (*p* < 0.001). Viable cell counts were significantly lower for the efinaconazole 10% solution compared to the tavaborole 5% solution (*p* < 0.001) and ciclopirox 8% nail lacquer (*p* < 0.001). No significant difference was observed in viable cell counts between the tavaborole 5% solution and ciclopirox 8% nail lacquer.

## 4. Discussion

Since dermatophytes are mainly present under a densely keratinized nail plate in onychomycosis, the route of entry into the nail bed plays a vital role in determining the efficacy of a drug. Oral antifungals can reach the nail bed by achieving antifungal level via the bloodstream. On the other hand, the primary route of drug delivery for topical antifungal is transungual, and difficulties are associated with successfully treating the disease with topical antifungals. In order to be effective, topical antifungals must penetrate the nail plate and retain their antifungal activity in the nail bed, both of which are affected by their binding to keratin [17,18]. Two effective topical antifungals, efinaconazole and tavaborole, have recently become available for the treatment of onychomycosis in the USA. Both have lower keratin affinities [10,19], and greater human nail permeations, than amorolfine or ciclopirox [10,12,19].

The activity of topical antifungals has been evaluated based on their MICs, measured using the microdilution method with MOPS-buffered RPMI 1640 medium. However, antifungals are deactivated by binding to keratin in the keratin-rich environment of the nail plate or nail bed [17,18]. We previously reported an assay system of fungicidal activity using keratin-containing medium that mimics this keratin-rich environment [10], and enables antifungal potency in the nail plate and nail bed to be evaluated.

In the present study, we compared fungicidal activities in the presence of keratin and MICs of three antifungals (efinaconazole, tavaborole, and ciclopirox) in order to identify important factors that may contribute to in vivo efficacy.

MIC ranges, MIC_50_ and MIC_90_ values for efinaconazole, tavaborole, and ciclopirox for *T. rubrum* and *T. mentagrophytes* suggested that efinaconazole was more active than tavaborole and ciclopirox.

In order to estimate antifungal potencies in the nail plate and nail bed, their fungicidal activity in the presence of keratin was evaluated. Efinaconazole exhibited potent fungicidal activity against *T. mentagrophytes* at 5 μg/mL in a time-dependent manner, whereas tavaborole showed only fungistatic activity, even at 20 μg/mL, and ciclopirox was not active. The reasons for which ciclopirox shows no antifungal activity in keratin-containing medium may be attributed to its MIC for *T. mentagrophytes* being higher than that of efinaconazole, and its having a higher keratin affinity than efinaconazole [10], and tavaborole [20]. A possible reason for the weaker effect of tavaborole could also be its higher MIC compared with efinaconazole.

The in vivo efficacy of efinaconazole 10% solution, tavaborole 5% solution, and ciclopirox 8% nail lacquer were evaluated in a guinea pig onychomycosis model. The results obtained showed all three antifungals significantly decreased viable cell counts. Viable cell counts were significantly lower with efinaconazole 10% solution compared to tavaborole 5% solution and ciclopirox 8% nail lacquer. No significant difference was observed in viable cell counts between the tavaborole 5% solution and ciclopirox 8% nail lacquer groups. A comparison of the results of the in vitro and in vivo experiments suggests that the superior in vivo efficacy of efinaconazole was due to its lower MIC and potent fungicidal activity in the presence of keratin. Although ciclopirox was not active in keratin-containing medium, the drug showed therapeutic efficacy in the onychomycosis model, which suggests that its MIC contributed to in vivo efficacy. On the other hand, tavaborole has the highest MIC and weak fungicidal action, but showed therapeutic efficacy in the onychomycosis model, perhaps due to its higher nail penetration [12,19].

The results we found in our in vivo studies are consistent with the relative mycological and complete cure rates reported in clinical trials [5,7,8] (Table 2). Matsuda et al. have previously reported that the efficacy coefficient, the ratio of drug nail permeation to MIC for *T. rubrum* in the presence of keratin, is useful for predicting the clinical efficacy of topical antifungal drugs [14]. The results of the present study suggest that fungicidal activity in the presence of keratin is also an important factor contributing to the in vivo efficacy of topical antifungal drugs.

In conclusion, not only low MIC, but also potent fungicidal activity in the presence of keratin, are required for topical antifungal drugs to exert high in vivo efficacy. This finding will help strategies for developing more effective topical antifungal drugs for the treatment of onychomycosis in the future.

## Figures and Tables

**Table 1:** MIC range, MIC_50_, and MIC_90_ of efinaconazole, tavaborole, and ciclopirox for *T. rubrum* and *T. mentagrophytes.*

Test Substances	MIC (μg/mL)					
	*T. rubrum* (10 Strains)			*T. mentagrophytes* (10 Strains)		
	Range	MIC_50_	MIC_90_	Range	MIC_50_	MIC_90_
Efinaconazole	0.0020–0.0078	0.0039	0.0078	0.0039–0.031	0.0078	0.016
Tavaborole	4.0–8.0	8.0	8.0	4.0–8.0	4.0	8.0
Ciclopirox	0.25–1.0	0.50	0.50	0.50	0.50	0.50

**Table 2:** Collective table of mycological and complete cure rates of efinaconazole 10% solution, tavaborole 5% solution, and ciclopirox 8% nail lacquer in clinical trials for onychomycosis.

Drugs	Mycological Cure Rate (%)	Complete Cure Rate (%)	Reference
Efinaconazole 10% solution	55.2, 53.4	17.8, 15.2	[7]
Tavaborole 5% solution	31.1, 35.9	6.5, 9.1	[8]
Ciclopirox 8% nail lacquer	29, 36	5.5, 8.5	[6]
